# Barriers to Using Breast Cancer Screening Methods Among Adult Females in Jeddah, Saudi Arabia: A Cross-Sectional Study

**DOI:** 10.7759/cureus.41739

**Published:** 2023-07-11

**Authors:** Marwan Bakarman, Duha Kalthoum, Iman Wahby Salem, Razan O Alshuaibi, Thikra A Almohammadi, Rana A Beser, Raghad H Almuwallad, Leena A Alotaibi

**Affiliations:** 1 Medicine, King Abdulaziz University Faculty of Medicine, Rabigh, SAU; 2 Community and Family Medicine, King Abdulaziz University Faculty of Medicine, Jeddah, SAU; 3 Community Medicine, King Abdulaziz University Faculty of Medicine, Jeddah, SAU; 4 Medicine, King Abdulaziz University, Rabigh, Jeddah, SAU; 5 Medicine, King Abdulaziz University Faculty of Medicine, Jeddah, SAU; 6 Medicine, King Abdulaziz University Hospital, Jeddah, SAU; 7 Otolaryngology - Head and Neck Surgery, King Abdulaziz University Faculty of Medicine, Jeddah, SAU

**Keywords:** bse, screening mammogram, breast cancer screening barriers, breast cancer screening, breast cancer

## Abstract

Introduction

Breast cancer (BC) is one of the most common cancers worldwide and it considerably increases morbidity and mortality globally. Screening methods, such as self-examination, clinical examination, and mammography, can help in early detection and treatment, which will help in improving survival rates and reducing mortality. While regular screening of the breast is essential to detect the earliest stages of breast cancer, not all women adhere to regular breast screening.

Methods

A cross-sectional study was conducted in Jeddah, Saudi Arabia, between December 2021 to July 2022. using an online self-administered questionnaire. The total number of responses (n = 328), Data was analyzed using SPSS 25.

Results

In this study, out of the 328 respondents, 18.9% reported undergoing regular mammography, 14.3% reported having regular clinical breast examinations, and 38.1% reported practicing regular breast self-examinations.. In addition, the participants' most known warning signs of BC were a lump under their armpit (69.1%). the most perceived barrier to breast self-examination (BSE) was Doing a breast examination will make her worry about what is wrong with her breast (47%). whereas the most barrier to clinical breast examination (CBE) was embarrassment (45.9%). On the other hand, the main barriers that prevented women from having mammograms were embarrassment (36%) and pain (32.6%).

Conclusion

The most perceived barrier to BSE was women’s concern, while embarrassment and painful procedures were significant barriers to performing mammography and CBE. Therefore, adult females in Jeddah need educational programs to improve their knowledge and increase public awareness of breast cancer screening for early detection.

## Introduction

Breast cancer (BC) has surpassed lung cancer as the most commonly diagnosed cancer among women, with an estimated 2.3 million new cases, accounting for approximately 11.7% of all cancer diagnoses. [1][2]. While BC is more prevalent in developed countries compared to developing countries, mortality rates are notably higher in the latter, representing around 45% of newly reported cases and contributes to approximately 54% of the total annual fatalities [3][4][5]. According to the world health organization (WHO), BC affects approximately 2 million women annually. In 2020, over 3,500 women in Saudi Arabia were diagnosed with BC, with an estimated mortality rate of 20.4% [1]. Several risk factors contribute to breast cancer, ranging from family history and genetic heritage to long-term use of hormone replacement therapy [6][7]. Early identification contributes to reducing breast cancer morbidity and achieve up to a 95% survival rate [8].

Breast self-examination (BSE), clinical breast examination (CBE), and mammography are three BC screening (BCS) techniques that are helpful in identifying the disease early [9]. Breast self-examination (BSE) is a simple, low-cost, and effective method. It can be performed at home increases the chance of treatment and women’s survival rate [10]. Despite the relative advantages of (BSE) and breast cancer screening tools, their use is still relatively underrepresented in the Kingdom of Saudi Arabia, a nation with free healthcare [11]. Clinical breast examination (CBE), carried out in a clinic by a qualified doctor or healthcare practitioner, is a crucial tool for areas where residents have limited access to expensive technical services [12]. Mammography, on the other hand, is an advanced screening method that uses X-ray images to detect BC [13]. Mammography is an effective screening tool for reducing breast cancer mortality in women aged 40 and above, but it is important to acknowledge potential drawbacks such as the emotional impact of the procedure and women's concerns about mammography results, which should be taken into account when making decisions about screening [14,15].

Even while routine breast screening is necessary to identify BC in its initial stages, not all women do it [9]. According to the 2015 National Saudi Health Interview Survey, only 1,135 out of 10,735 women underwent BC screenings, which indicates that women have an extremely low screening rate [16]. The low uptake of mammography among Saudi women can be attributed to limited knowledge and awareness about breast cancer, traditional beliefs and cultural norms that discourage seeking preventive medical exams, perceptions of health services that may lack female providers and information, limited access to female physicians in specialized healthcare facilities, and cultural barriers and gender roles [16]. While these factors contribute to the low screening rates, it is important to conduct further studies to fully understand the range of barriers and challenges faced by Saudi women in accessing and utilizing breast cancer screening services. Women, especially those of younger age, should be educated about the advantages of early BC diagnosis so that they will be ready to take the necessary examinations once they reach the required examination age. Lack of knowledge of the advantages of BC screening methods is one of the most cited reasons given for neglecting screening procedures [17]. According to another article, women avoid getting screened for BC most frequently because they assume they don't have the disease and don't need to be [18]. Additionally, a Saudi study discovered that women in Saudi Arabia avoid mammograms most frequently because they believe they are unnecessary, followed by their worries about the outcomes of mammograms [14].

Previous studies have identified various factors deterring women from participating in BC screening, such as emotional issues, lack of awareness about the consequences, and avoidance of the process [11,19]. Difficulty in scheduling appointments with doctors and concerns about being diagnosed with BC have also been reported as reasons for women [20]. Additionally, limited access to mammography services and long travel distances particularly affects women in rural areas [21]. To address these barriers, a comprehensive approach involving healthcare providers and policymakers is crucial. Strategies include increasing education and awareness through campaigns, developing culturally sensitive messaging, improving access to female healthcare providers and specialized facilities, collaborating with community leaders, establishing support groups, and counseling services, implementing policy incentives and reforms, and fostering collaboration and partnerships among stakeholders.

The barriers to BC screening for different screening techniques among women in the city of Jeddah were not considered in earlier studies of comparable scale. The purpose of this paper is to understand some of the social, physical, and cultural barriers that are playing a role in assisting early detection, preventive measurements, and reducing the incidence of BC among women in Jeddah, Saudi Arabia, 2021.

## Materials and methods

This is an analytic cross-sectional study aimed to assess the barriers to using different screening methods for breast cancer among adult females in Jeddah, Saudi Arabia, in 2021. This study was approved by the institutional review board of King Abdulaziz University (Reference No 29-22). Between December 2021 and July 2022, an online survey was conducted among adult women aged 20 years and above residing in Jeddah. The survey was distributed via popular social media platforms such as WhatsApp and Snapchat, utilizing publicly listed phone numbers obtained from a national telecom company. Participant confidentiality and anonymity were ensured, and the online format allowed for private responses. The questionnaire used neutral language and employed snowball sampling to gather diverse participant responses. Initially, 345 responses were received, but after excluding individuals who did not meet the criteria of being adult women aged 20 years or older residing in Jeddah, we obtained a final sample size of 328 responses out of the calculated sample size of n=377, resulting in a response rate of 87%. The sample size calculation was done using Rao software with a 95% confidence interval and a 5% margin of error based on the population size (Raosoft, Inc., Seattle, USA).

The survey used in this study was obtained from a previous research project without modifications [22]. To ensure accurate translation, the survey was professionally translated into Arabic by a translation company. Additionally, the survey underwent validation by two epidemiologists and one internal medicine specialist to ensure its reliability and validity. The survey consisted of three sections. The first section included questions related to participants' demographics, including age, education level, employment, and marital status. In the second section, participants were queried about their personal experience with breast cancer, as well as whether they had a family member or friend who had been diagnosed with the disease. This section also assessed participants' knowledge regarding breast cancer, including familiarity with warning signs, risk factors, and their engagement in breast cancer screening practices, either through self-examination or by seeking medical assistance.

The third section contained the Modified Champion Health Belief Model Scale (CHBMS) which is a valid and reliable tool to measure susceptibility, seriousness, barriers to BSE, barriers to CBE, and mammography barriers, as well as confidence in BSE efficacy and health motivation [23]. Participants completed the CHBMS questionnaire in Arabic, and the construct validity was evaluated through factor analysis with varimax rotation. A loading criterion of .45 was applied during the analysis. The final Arabic version of the CHBMS (CHBMS-A) comprised 22 items distributed among five sub-scales: susceptibility (two items), seriousness (two items), barriers (11 items), confidence (three items), and motivation (three items). The reliability of the CHBMS-A was demonstrated by Cronbach's alpha coefficients, which ranged from .91 to .79, indicating good internal consistency. Overall, the CHBMS-A exhibited reliability and validity for assessing beliefs and practices related to breast cancer screening among Arab women, making it suitable for use in interventions targeting this population.

Statistical analyses were done using IBM SPSS Statistics for Windows software version 26 (IBM Corp., Armonk, NY) to test and evaluate the hypothesis. Descriptive statics of frequency tables, cross-tabulation, and percentages were constructed using the software. The relationships between performing BSE on a regular basis and participants' age, marital status and educational level were conducted using the Chi-squared test (χ2). Furthermore, multivariate logistic regression analysis of the barriers to BCS was done with a 95% CI to assess the risk factors (independent predictors). A p-value of less than 0.05 was considered statistically significant.

## Results

A total of 328 women took part in this study. Most participants (55.2%) had an age ≥45 years, 69.8% were married, and 79.6% had a university level of education and above. Of the participants, 46.3% had a family member or friend who experienced breast cancer and only 5.8% had ever been diagnosed with breast cancer (Table 1). 

**Table 1:** Distribution of participants according to demographics, history of breast cancer, and BCS practices (N=328) BCS: breast cancer screening; BC: breast cancer

Variable	Categories	Frequency (n)	Percentage (%)
Age	20-29 years	65	19.8
	30-39 years	31	9.5
	40-44 years	51	15.5
	≥ 45 years	181	55.2
Marital status	Divorced	23	7
	Married	229	69.8
	Single	67	20.4
	Widower	9	2.7
Educational level	Illiterate	2	0.6
	Intermediate	5	1.5
	Primary	2	0.6
	Secondary	58	1.7
	University and above	261	79.6
History of BC among family or friends	Yes	152	46.3
	No	176	53.7
History of BC diagnosis	Yes	19	5.8
	No	309	94.2
Undergoing mammography on a regular basis	Yes	62	18.9
	No	266	81.1
Having a clinical breast examination on a regular basis	Yes	47	14.3
	No	281	85.7
Having a breast self-examination on a regular basis	Yes	125	38.1
	No	203	61.9

Knowledge of BC and risk factors of BC

**Figure 1:**
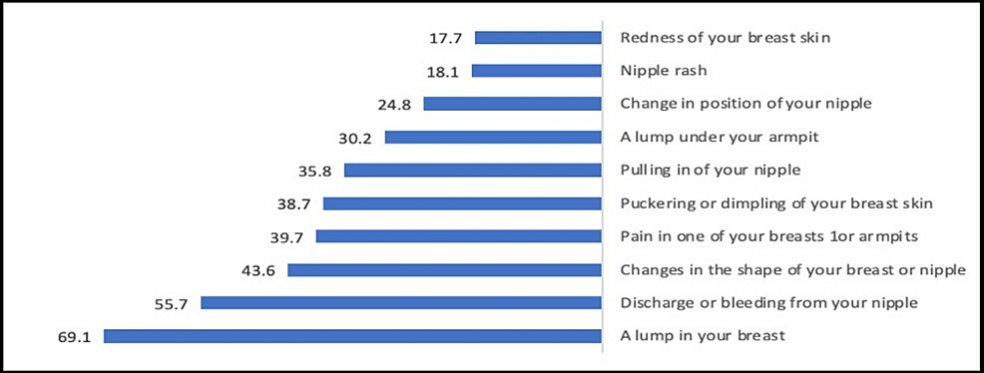
Percentage distribution of the participants according to their knowledge about warning signs of breast cancer

**Figure 2:**
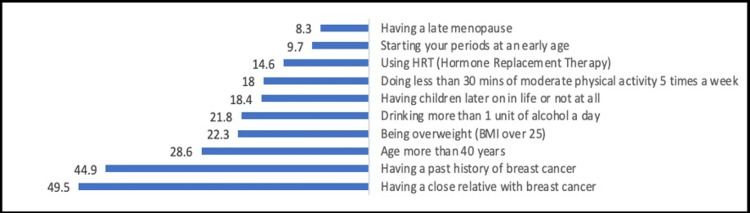
Percentage distribution of the participants according to their knowledge about the risk factors of breast cancer

The most known warning signs of BC by the participants were a lump in your breast (69.1%), discharge or bleeding from the nipple (55.7%) and changes in the shape of the breast or nipple (43.6%). And the most known risk factors of BC were having a close relative with breast cancer (49.5%) and having a history of breast cancer 44.9% (Figures 1, 2).

Table 2 presents the participants' responses to the Modified Champion Health Belief Model Scale (CHBMS) questionnaire items. Of them, 14.3% reported being likely to get breast cancer and 4.3% reported having a great chance to get BC in the next few years. More than half (50.3%) thought that BC is scary and thought that if someone had breast cancer, her whole life would change. As for barriers to BSE, 47% thought that doing BSE will make them worry about what is wrong with their breast, 17.4% thought that BSE takes too much time and 37.2% said that it is hard to remember to do BSE. Regarding CBE barriers, 45.9% said that it is embarrassing for them to have a breast exam by a physician, 34.5% said that breast exams by a physician can be painful, 18.9% were afraid of not being able to go to a physician for breast exam and 24.1% said that it is time-consuming. The most common barriers to mammography were being too embarrassing (36%) and being too painful (32.6%). As for the participants' confidence in BSE efficacy, 44.5% reported that they could find a breast lump by performing BSE, 56.4% said that they are able to tell something is wrong with their breast when they looked in the mirror, and 39.6% said that they can perform BSE correctly. Health motivations were exercising at least three times per week (31.4%), eating well-balanced meals (47.3%), and maintaining good health is extremely important to them (80.5%). 

**Table 2:** Participants' response to the modified champion health belief model questionnaire items BSE: breast self-examination; CBE: clinical breast examination

Modified Champion Health Belief Model Scale Questionnaire			
Concept	Agree n (%)	Neutral n (%)	Disagree n (%)
Susceptibility It is likely that I will get breast cancer	47 (14.3)	155 (47.3)	126 (38.4)
My chances of getting breast cancer in the next few years are great	14 (4.3)	154 (47)	160 (48.8)
Seriousness The thought of breast cancer scares me	15 (50.3)	82 (25)	81 (24.7)
If someone had breast cancer, her whole life would change	165 (50.3)	90 (27.4)	73 (22.3)
BSE barriers to doing breast examination will make me worry about what is wrong with my breast	154 (47)	73 (22.3)	101 (30.8)
BSE takes too much time	57 (17.4)	104 (31.7)	167 (50.9)
It is hard to remember to do a breast examination	122 (37.2)	107 (32.6)	99 (30.2)
CBE barriers It is embarrassing for me to have a breast exam performed by a physician	149 (45.9)	70 (21.3)	109 (33.2)
Breast exams performed by a physician can be painful	113 (34.5)	97 (29.6)	118 (36)
I am afraid I would not be able to go for a breast exam performed by a physician.	62 (18.9)	76 (23.2)	190 (57.9)
Breast exam performed by a physician is time-consuming	79 (24.1)	105 (32)	144 (43.9)
Mammography barriers I have other problems more important than getting a mammogram	58 (17.7)	93 (28.4)	177 (54)
Having a mammogram is too painful	107 (32.6)	106 (32.3)	115 (35.1)
I am afraid I would not be able to go to the mammogram appointment	57 (17.4)	80 (24.4)	191 (58.2)
Having a mammogram is too embarrassing	118 (36)	74 (22.6)	136 (41.5)
Having a mammogram takes too much time	77 (23.5)	111 (33.8)	140 (42.7)
Confidence in BSE efficacy I could find a breast lump by performing BSE	1146 (44.5)	119 (36.3)	63 (19.2)
I am able to tell something is wrong with my breast when I look in the mirror	158 (56.4)	91 (27.7)	52 (15.9)
I can perform BSE correctly	130 (39.6)	23 (37.5)	75 (22.9)
Health motivation I exercise at least 3 times/week	103 (31.4)	88 (26.8)	137 (41.8)
I eat well-balanced meals	155 (47.3)	12 (34.1)	61 (18.6)
Maintaining good health is extremely important to me	264 (80.5)	51 (15.5)	13 (4)

Table ) shows that participants who underwent mammography or clinical breast examination on a regular basis had a significantly higher percentage of having age ≥45 years and being married (p=<0.05). On the other hand, a non-significant relationship was found between undergoing mammography or clinical breast examination on a regular basis and participants' educational level (p=>0.05).

**Table 3:** Relationship between mammograms and routine clinical breast examinations, and some participants' demographic factors (n=328) *p-value is statistically significant

Variable	Undergo mammography on a regular basis				χ2 value p-value	Have clinical breast examination on a regular basis				χ2 value p-value
	No		Yes			No		Yes		
	(n)	%	(n)	%		(n)	%	(n)	%	
Age					28.97 <0.00*					21.52 <0.00*
20-29	65	24.4	0	0.0		64	22.8	1	2.1	
30-39	27	10.2	4	6.5		30	10.7	1	2.1	
40-44	45	16.9	6	7.9		46	16.4	5	10.6	
≥ 45	129	48.5	52	83.9		141	50.2	40	85.1	
Marital status					10.57 0.01*					14.17 0.00*
Divorced	20	7.5	3	4.8		23	8.2	0	0.0	
Married	176	66.2	53	85.5		188	66.9	41	87.2	
Single	63	22.7	4	6.5		64	22.8	3	6.4	
Widower	7	2.6	2	3.2		6	2.1	3	6.4	
Educational level					7.03 0.13					1.99 0.73
Illiterate	1	0.4	1	1.6		2	0.7	0	0.0	
Intermediate	3	1.1	2	3.2		4	1.4	1	2.1	
Primary	2	0.8	0	0.0		2	0.7	0	0.0	
Secondary	42	15.8	16	25.8		47	16.7	11	19	
University and above	218	82	43	69.4		226	80.4	35	74.5	

Multivariate logistic regression analysis was done to assess the risk factors (independent predictors) of having any barrier to breast examination (Table 4). It was found that the odds of having barriers to BSE, CBE, or mammography were slightly higher among married women, older age, and having a university level of education. Yet participants' age, marital status, or educational level were not independent predictors (p=>0.05). It was found that being diagnosed with breast cancer significantly increased the odds of undergoing mammography (OR 12.13 (95% CI: 3.8-38.67) p=<0.001) but this is expected as women with breast cancer diagnosis have to undergo mammography pre- and post-diagnosis. Furthermore, having an age of ≥45 years and a university level of education or above statistically increases the odds of undergoing mammography (OR 2.64 (95% CI: 1.65-4.22), OR 0.6 (95% CI: 0.36-0.97), respectively, p=<0.05). A previous diagnosis of breast cancer statistically increased the odds of undergoing CBE regularly (OR 7.4 (95% CI: 2.35-23.2), p=<0.005). Age ≥45 years also statistically increased the odds of getting CBE regularly (p=<0.05). Regarding having a BSE on a regular basis, it was found that being at an age of 45 years and above, education level of university degree or higher, as well as being diagnosed with breast cancer were significant predictors (p=<0.05). 

**Table 4:** Multivariate logistic regression analysis of risk factors of having any barrier to BSE, CBE, or mammography on a regular basis BSE, breast cancer screening; CBE, clinical breast examination *p-values <0.05 for odds of having any barrier to BSE, CBE, or mammography

Variable	Having any barrier to BSE, CBE, or mammography		
	P-value*	Odds Ratio	Upper and lower (CI:95%)
Age	0.926	0.98	(0.68-1.61)
Marital status	0.854	0.93	(0.44-1.97)
Educational level	0.675	0.86	(0.45-1.57)
Do you have a family member or friend who experienced breast cancer?	0.339	0.67	(0.29-1.51)
Have you ever been diagnosed with breast cancer?	0.723	0.68	(0.08-5.53)
	Undergoing mammography on a regular basis		
Age	<0.001	2.64	(1.65-4.22)
Marital status	0.428	1.25	(0.71-2.2)
Educational level	0.04	0.6	(0.36-0.97)
Do you have a family member or friend who experienced breast cancer?	0.377	1.33	(0.72-3.8)
Have you ever been diagnosed with breast cancer?	<0.001	12.13	(3.8-38.67)
	Undergoing clinical breast examination on a regular basis		
Age	0.001	2.79	(1.54-5.05)
Marital status	0.074	1.75	(0.94-3.24)
Educational level	0.602	0.84	(0.43-1.61)
Do you have a family member or friend experienced breast cancer?	0.196	1.66	(0.76-3.62)
Have you ever been diagnosed with breast cancer?	0.001	7.4	(2.35-23.2)
	Having a breast self-examination on a regular basis		
Age	0.006	1.42	(1.1-1.82)
Marital status	0.576	1.41	(0.7-1.84)
Educational level	0.025	2	(1.09-3.67)
Do you have a family member or friend experienced breast cancer?	0.099	1.56	(0.91-2.66)
Have you ever been diagnosed with breast cancer?	0.047	3.17	(1.01-9.91)

## Discussion

The aim of this study was to assess the level of knowledge of breast cancer and screening measures as well as some of the sociodemographic barriers that play into the decision of screening for BC among women in Jeddah, Saudi Arabia. Participants had a good level of understanding of BC, including its symptoms and risk factors. However, practising BCS was low for self-examination, clinical screening, and mammography, which curbs early detection and recovery. This is in line with similar studies in the literature [11,18].

Breast cancer screening is provided free of charge to eligible females in Saudi Arabia within the recommended screening age group [14]. It plays a crucial role in the early detection of breast cancer, leading to a decrease in both morbidity and mortality rates associated with the disease [22]. However, the findings of this study indicate a low performance rate for various breast cancer screening methods. Similar results were reported in a previous study conducted among 358 Omani women, which showed that less than a quarter of women had not received BC screening [18]. Additionally, a study done by Azaiza et al among 397 women in 2010 showed that the majority had never received mammography or CBE [24]. Therefore, this research may encourage healthcare practitioners to advise women to use breast cancer screening to reduce the prevalence of BC.

We used the CHBMS questionnaire to investigate barriers to BCS among women. The factors that influence BSE behavior include barriers, health motivation, perceived benefits of BSE, and susceptibility. Most participants were aware of how serious BC is, so fear and anxiety were common themes and screening for BC is worrisome, so most women avoid it. While self-confidence in the ability to perform BSE was high, most participants cannot remember to periodically do the examination and felt embarrassed to have the doctor examine them and/or do mammography. This finding is consistent with similar studies in the literature [18-22]. A study done by Azaiza et al found that nearly half of their participants were embarrassed when undergoing an examination [24]. Despite most of their participants knowing the proper technique of BSE, 83.8% of them had not undergone BCS. As a result, there is a possibility that this will interfere with the early detection of disease and reduce the likelihood of a cure [22].

Regarding preventive practices to detect BC, one of the key findings is that having BCE is statistically dependent on age above 45 and a history of BC diagnosis. Furthermore, conducting a self-examination was also statically dependent on age above 45, having a history of breast cancer, and higher levels of education. Furthermore, education was also a strong factor, along with age and previous history of BC diagnosis, for women to go under mammographic examination regularly, which is in line with a previous study conducted in Saudi Arabia by Al-Wassia et al. Education level was found to be strongly associated with higher mammography knowledge [14]. In contrast, the same study revealed that women with a positive family history of BC reported utilizing mammography at a lower rate than those without such a history [14]. Therefore, turning the current BC screening in Saudi Arabia into organized programs will enhance the screening rate and lower the disease's cost burden [16].

The study has several limitations. First, the use of the snowball sampling method carries a significant risk of selection bias. The reliance on participants to refer others from their social networks may introduce a non-random element into the sample, potentially excluding individuals who are less connected or have different characteristics. Therefore, caution should be exercised when generalizing the findings to the broader population of adult women in Jeddah, or other regions in Saudi Arabia. Second, although various measures were implemented to control for social desirability bias, such as ensuring anonymity, conducting the survey online, and using neutral language, it is important to recognize that complete elimination of this bias is challenging, and self-reported data may still be influenced by it. Third, there is a possibility of reporting bias since the data relied on self-reports. Fourth, as the data was cross-sectional, causality cannot be established, and caution is advised when interpreting the results. Lastly, while this study examined the factors influencing breast cancer screening uptake among adult women in Jeddah, Saudi Arabia, it is important to acknowledge that certain key determinants were not specifically investigated. Factors such as the quality of screening services, the skill level of healthcare providers, cultural beliefs surrounding breast cancer screening, and the availability of social support for women were not explored in depth. Future research should focus on examining these aspects to better understand their influence on screening uptake and to develop targeted interventions that address these crucial determinants.

## Conclusions

While the level of knowledge regarding BC among women in this study was high, utilization of different screening techniques, despite their availability, was low. To address this, a comprehensive approach is needed. Women should be educated about the benefits of breast cancer screening and encouraged to undergo it. Initiatives should focus on improving women's comfort during screening. Additionally, assessing the quality of breast cancer screening services in Saudi Arabia is important to ensure they meet global standards. Collaboration among healthcare providers, policymakers, and stakeholders is crucial to develop and implement comprehensive screening programs that target emotional barriers and avoidance.

## References

[1] Sung H, Ferlay J, Siegel RL, Laversanne M, Soerjomataram I, Jemal A, Bray F (2021). Global Cancer Statistics 2020: GLOBOCAN Estimates of Incidence and Mortality Worldwide for 36 Cancers in 185 Countries. CA Cancer J Clin.

[2] Okoronkwo IL, Ejike-Okoye P, Chinweuba AU, Nwaneri AC (2015). Financial barriers to utilization of screening and treatment services for breast cancer: an equity analysis in Nigeria. Niger J Clin Pract.

[3] Shepardson LB, Dean L (2020). Current controversies in breast cancer screening. Semin Oncol.

[4] I World Health Organization. International Agency for Research on Cancer Latest world cancer statistics: global cancer burden rises to 14.1 million new cases in 2012: marked increase in breast cancers must be addressed. https://www.iarc.who.int/wp-content/uploads/2018/07/pr223_E.pdf.

[5] Oeffinger KC, Fontham ET, Etzioni R (2015). Breast Cancer Screening for Women at Average Risk: 2015 Guideline Update From the American Cancer Society. JAMA.

[6] Martin AM, Weber BL (2000). Genetic and hormonal risk factors in breast cancer. J Natl Cancer Inst.

[7] Albrektsen G, Heuch I, Hansen S, Kvåle G (2005). Breast cancer risk by age at birth, time since birth and time intervals between births: exploring interaction effects. Br J Cancer.

[8] Yarbrough SS, Braden CJ (2001). Utility of health belief model as a guide for explaining or predicting breast cancer screening behaviours. J Adv Nurs.

[9] (2006). World Health Organization, World Health Organization. Reproductive Health, World Health Organization. Chronic Diseases, Health Promotion. Comprehensive cervical cancer control: a guide to essential practice. World Health Organization.

[10] Bretthauer M, Kalager M (2013). Principles, effectiveness and caveats in screening for cancer. Br J Surg.

[11] Gonzales A, Alzaatreh M, Mari M, A Saleh A, Alloubani A (2018). Beliefs and Behavior of Saudi Women in the University of Tabuk Toward Breast Self Examination Practice. Asian Pac J Cancer Prev.

[12] Veitch D, Goossens R, Owen H, Veitch J, Molenbroek J, Bochner M (2019). Evaluation of conventional training in Clinical Breast Examination (CBE). Work.

[13] Olsen O, Gøtzsche PC (2001). Screening for breast cancer with mammography. Cochrane Database Syst Rev.

[14] Al-Wassia RK, Farsi NJ, Merdad LA, Hagi SK (2017). Patterns, knowledge, and barriers of mammography use among women in Saudi Arabia. Saudi Med J.

[15] Uk I. Cancer, B.(2012 B.(2012 The benefits and harms of breast cancer screening: an independent review. Lancet. 380.

[16] El Bcheraoui C, Basulaiman M, Wilson S (2015). Breast cancer screening in Saudi Arabia: free but almost no takers. PLoS One.

[17] Ferdous M, Goopy S, Yang H, Rumana N, Abedin T, Turin TC (2020). Barriers to Breast Cancer Screening Among Immigrant Populations in Canada. J Immigr Minor Health.

[18] Al-Azri M, Al-Rubaie K, Al-Ghafri S, Al-Hinai M, Murthi Panchatcharam S (2020). Barriers and Attitudes toward Breast Cancer Screening among Omani Women. Asian Pac J Cancer Prev.

[19] Consedine NS, Magai C, Neugut AI (2004). The contribution of emotional characteristics to breast cancer screening among women from six ethnic groups. Prev Med.

[20] Al-Khamis NK (2018). Low Awareness of Breast Cancer and Considerable Barriers to Early Presentation Among Saudi Women at a Primary Care Setting. J Cancer Educ.

[21] Khan-Gates JA, Ersek JL, Eberth JM, Adams SA, Pruitt SL (2015). Geographic Access to Mammography and Its Relationship to Breast Cancer Screening and Stage at Diagnosis: A Systematic Review. Womens Health Issues.

[22] Al-Ghadeer B, Al-Ghadeer A, Aldoughan S (2021). Breast cancer knowledge and barriers to screening among women in Al-Ahsa, Saudi Arabia. Int J Med Dev Ctries.

[23] Champion VL (1999). Revised susceptibility, benefits, and barriers scale for mammography screening. Res Nurs Health.

[24] Azaiza F, Cohen M, Awad M, Daoud F (2010). Factors associated with low screening for breast cancer in the Palestinian Authority: relations of availability, environmental barriers, and cancer-related fatalism. Cancer.

